# Fragmented α-Amylase into Microporous Metal-Organic Frameworks as Bioreactors

**DOI:** 10.3390/ma14040870

**Published:** 2021-02-11

**Authors:** Li-Hao Liu, Ru-Yin Chiu, Pamela Berilyn So, Stephen Lirio, Hsi-Ya Huang, Wan-Ling Liu, Chia-Her Lin

**Affiliations:** 1 Department of Chemistry, Chung Yuan Christian University, Taoyuan 32023, Taiwan; leedorins@gmail.com (L.-H.L.); jo6yplum@gmail.com (R.-Y.C.); pbtiuso@gmail.com (P.B.S.); stephenblirio@gmail.com (S.L.); starsea800@hotmail.com (W.-L.L.); 2 Department of Chemistry, National Taiwan Normal University, Taipei 11676, Taiwan

**Keywords:** metal-organic framework, enzyme immobilization, catalysis, bioreactor, size matching

## Abstract

This work presents an efficient and facile strategy to prepare an α-amylase bioreactor. As enzymes are quite large to be immobilized inside metal-organic frameworks (MOFs), the tertiary and quaternary structures of α-amylase were first disrupted using a combination of urea, dithiothreitol (DTT), and iodoacetamide (IAA). After losing its tertiary structure, the unfolded proteins can now penetrate into the microporous MOFs, affording fragmented α-amylase@MOF bioreactors. Among the different MOFs evaluated, UiO-66 gave the most promising potential due to the size-matching effect of the α-helix of the fragmented α-amylase with the pore size of UiO-66. The prepared bioreactor exhibited high yields of small carbohydrate (maltose) even when reused up to 15 times (>80% conversion).

## 1. Introduction

Biocatalysis is a chemical process that utilizes natural catalysts, such as microorganisms or enzymes, toward the production of fine chemicals, which is pivotal in pharmaceutical and food industries [1]. Their excellent merits, including energy efficiency, minimal consumption of raw materials, as well as low waste and toxic side-products, are significant for the green chemistry approach. Biocatalysis is regarded as an attractive strategy to replace chemical synthetic routes in the laboratory, as well as in the industrial scale [2,3,4]. For economic reasons, recyclability is highly favorable for industrial application; thus, immobilization of enzymes on solid support has been regarded as an effective approach to circumvent this issue [5,6]. Immobilization techniques display enhanced interaction between the enzyme and matrix, or the containment of enzymes in a restricted space to anchor biocatalysts on solid supports. Moreover, immobilization may stabilize enzymes, especially upon exposure to harsh or drastic conditions. Besides, enzyme immobilization demonstrated to improve enzyme selectivity for specific reactions with less product contamination due to enzyme autolysis or proteolysis [7]. For years, sporadic examples have been reported about the enzyme immobilization through physical absorption [8,9] and chemical bonding [10], which are the conventionally used techniques.

Metal-organic frameworks (MOFs), one of the emerging nanomaterials of coordination-bonded networks consist of metal ions and organic ligands, have unique properties such as highly ordered crystallinity, large surface area, high porosity, tunable pore size, and high chemical and thermal stability [11,12,13]. To date, reports have demonstrated the potential of MOFs as solid support for enzymes and found them useful in biocatalysis. In general, enzymes are immobilized onto MOFs via physical adsorption [14,15], covalent bonding [16], dye tagging [17,18], and encapsulation [19,20,21,22,23,24] methods. However, MOFs usually carry microporous (<2 nm) windows, while most enzymes possess dimensions larger than 3 nm; thus, immobilization and eventual residence of these biomolecules in the MOFs are rather challenging and limited. As an alternative, our previous studies demonstrated the anchoring of small dye molecules (FITC—fluorescein isothiocyanate or NBD—4-chloro-7-nitrobenzofurazan, molecular size < 2 nm) onto larger biomolecules to form a host-guest complex, which were subsequently immobilized onto the microporous MOFs (MOFs’ micropores as host and dye as guest) to afford dye-tagged-enzyme@MOF bioreactors with high catalytic performance, reusability, and stability [17,18]. In the same way, we were also able to demonstrate the encapsulation of a small chiral molecule (L-proline) for the multipoint immobilization of lipase onto microporous MOFs, giving rise to a reusable and stable chiral catalyst [25]. Considering the diverse types and characteristics of commonly used enzymes for industrial application as well as the versatility of microporous MOFs, in this work, we sought to develop a simple strategy of immobilizing enzyme fragments into microporous MOFs materials for practical application. To make a useful comparison, the pristine form of enzyme was also immobilized onto the MOFs. Following our previous strategies, surface functionalization for the solid support was not necessary.

**Scheme 1:**
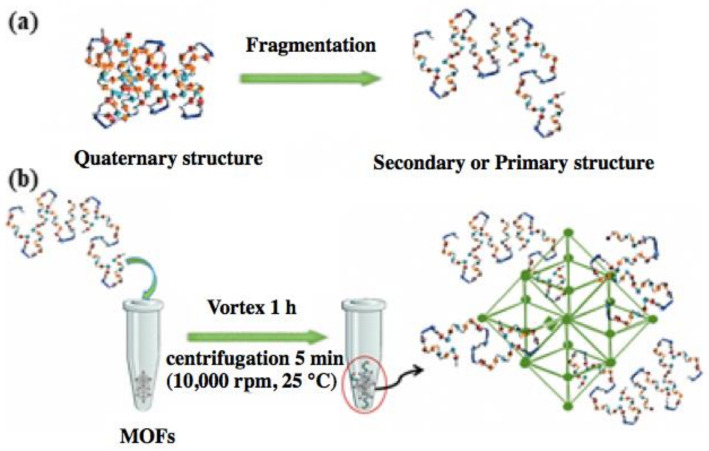
Schematic of (**a**) enzyme fragmentation process and (**b**) enzyme immobilization process.

Amylase is one of the most largely consumed industrial enzymes (~25% of the entire global enzyme market), which hydrolyzes starch to form maltose [26]. In particular, α-amylase (1,4-d-glucan-glucanhydrolase), a common amylase used for the production of high fructose corn syrup, ethanol, as well as in paper recycling [27], is used as a model enzyme to be immobilized into the MOF and applied as a bioreactor. Our strategy is to unravel the tertiary structure of the protein followed by the infiltration of the fragmented protein into the MOF pores. In this study, we hypothesize that the α-helix, a kind of secondary structure frequently occurring in proteins with a diameter about 0.6 nm [28], could enter into the MOF’s micropores. Here, a solution of α-amylase was fragmented using the combination of urea (to unfold the protein via hydrogen bonds), dithiothreitol (DTT) (to reduce the disulfide bridges), and iodoacetamide (IAA) (to methylate the reduced sulfide bonds and avoid the retrieval of disulfide bridges) [29] to completely disrupt the tertiary structure of α-amylase (Scheme 1a).

## 2. Materials and Methods

Material synthesis, characterization, and instrumentation parameters are given in detail in the supporting information.

### 2.1. Fragmentation Process for α-Amylase

The process followed the procedure of Kinter with slight modifications [30]. Briefly, 10 mg of α-amylase was dissolved in 1 mL aqueous solution composed of urea (6 M) and tris buffer (100 mM). Dithiothreitol (DTT) reducing reagent (200 mM, 5 μL) was then added to a 100 μL aliquot of the α-amylase solution (10 mg/mL) in a 1.5 mL plastic microcentrifuge tube, then vortex mixed. The protein mixture was allowed to stand at room temperature (RT) for 1 h. Then, 20 μL of the alkylating reagent, iodoacetamide (IAA, 200 mM), was added into the above solution and the alkylation process proceeded at RT in the dark for 1 h. To consume the unreacted IAA, another 20 μL of DTT reducing agent was added to the resulting solution with gentle vortex mixing, and the reaction was allowed to stand at RT for 1 h. Finally, the resulting solution was mixed with 775 μL of DI (deionized water) in order to reduce the urea concentration to ~0.6 M.

### 2.2. Immobilization Procedure

α-Amylase was immobilized onto the native MOFs (1 mg) by suspension in 100 μL of 1086 mg mL^−1^ fragmented α-amylase solution. The produced α-amylase immobilized MOFs (fragmented α-amylase@MOF) were washed twice with D.I. water (100 μL) prior to further hydrolysis and other tests.

### 2.3. Starch Hydrolysis via Fragmented α-Amylase@MOFs

Starch solution (100 μL, 0.25%) was added to the α-amylase@MOFs biocatalysts (1 mg) with gentle vortex mixing, then stirred for 30 min at 25 °C. The resulting digested product (25 μL) was mixed with 3,5-dinitrosalicyclic acid (DNS) reagent (500 μL) and then subjected to UV-visible spectrometric analysis.

### 2.4. Determination of α-Amylase Loading Capacity

The loading capacity of UiO-66 for fragmented α-amylase was evaluated by the fluorescence emission method. The difference between the fluorescence emission intensity of α-amylase solution before and after immobilization into UiO-66 was measured to evaluate the amount of α-amylase adsorbed on UiO-66.

## 3. Results and Discussion

Circular dichroism (CD) spectroscopy in the far-UV region revealed significant signal differences of repeating hydrogen-bonded α-helix structures (208 and 222 nm) [31] when α-amylase was processed for 1 h (Figure S1), indicating that it has lost its tertiary structures leading to the change in structural conformation. In addition, the fluorescence intensity emitted at 328 nm from tryptophan residues of α-amylase was observed to have a significant decrease on the fragmented α-amylase, which suggests the conformational changes in the protein [32] (Figure S2).

To probe the successful immobilization of the fragmented α-amylase with α-helix polypeptide chains (~0.6 nm diameter), we used UiO-66 with 0.7 nm pore as MOF support. Powder XRD patterns (Figure S3) confirmed that after immobilizing the fragmented α-amylase on UiO-66 (fragmented α-amylase@UiO-66), the ordered crystalline structure was retained and proved to be the same with the native UiO-66. Scanning electron microscope (SEM) images also showed similar cubic morphologies for both samples (as synthesized UiO-66 and fragmented α-amylase@UiO-66) (Figure S4). The fluorescence spectroscopy results for fragmented α-amylase@UiO-66 showed a minor shoulder signal at ~328 nm, which confirms that the fragmented α-amylase was immobilized successfully on UiO-66. Moreover, the emission band at ~386 nm due to the presence of UiO-66 was prominently enhanced (Figure S2). This observation suggests the presence of the interaction between α-amylase and UiO-66 cages. Furthermore, nitrogen adsorption/desorption measurement was used to determine changes in porosity on the solid support. Table S1 and Figure S5 summarize the surface areas of the samples. As shown, a significant decrease in the BET (Brunauer-Emmett-Teller) surface area was observed for the fragmented α-amylase@UiO-66 (96% decreases for fragmented α-amylase@UiO-66, Table S1).

To confirm the biocatalytic activity of the fragmented α-amylase, electrospray ionization-mass spectrometry (ESI-MS) was used to identify maltose [33], which is the major carbohydrate product of the random cleavage of starch, catalyzed by both the fragmented and native α-amylase (Figure S6). The results showed that the fragmented α-amylase still demonstrated its biological function similar to that of the native α-amylase. The relative hydrolytic activities of immobilized α-amylase were evaluated by determining the released reducing sugars from starch using 3,5-dinitrosalicyclic acid reagent (DNS) (referred as Bernfeld method) [34,35] (see supporting information). For microporous MOF substrates, UiO-66 was first used as solid support to immobilize native α-amylase and fragmented α-amylase, respectively, as depicted in Scheme 1b.

**Figure 1:**
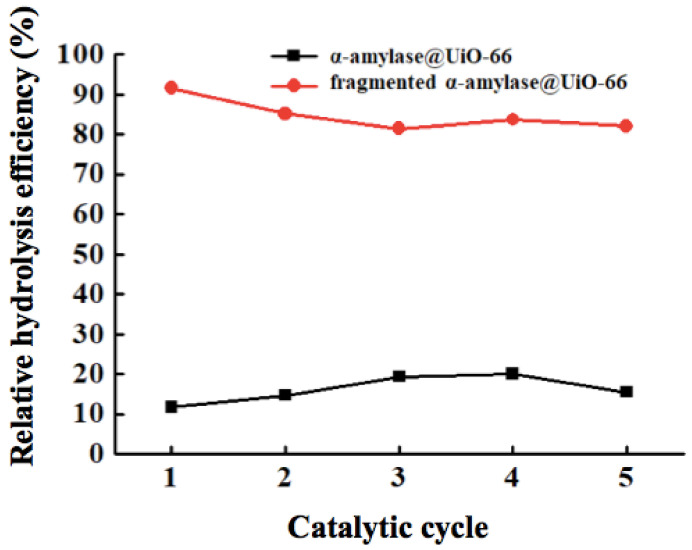
Comparison of the starch hydrolysis efficiency of native and fragmented α-amylase@UiO-66.

The results indicate the high catalytic stability of fragmented α-amylase@UiO-66 for starch hydrolysis even after 5 multiple cycles (maltose yield > 82%). Meanwhile, almost 11% maltose yield was obtained for the 1st catalytic cycle using the same solid support, UiO-66, as shown in Figure 1. This result is ascribed to size-matching effect between the α-helix of amylase and the window (or channel) of the MOFs. To probe this assumption, several MOFs (MIL-101(Cr), MIL-100(Al), and MIL-53(Al)) were used to immobilize the fragmented α-amylase. Among these MOFs, other than UiO-66, MIL-101(Cr) provided the highest stability after 5 catalytic cycles to hydrolyze starch with ~63% yield. In contrast, both MIL-100(Al) and MIL-53(Al) showed dramatic changes in their catalytic abilities after 3 cycles with 59% and 48% yields, respectively (Table 1 and Figure S7).

The superior catalytic activity of fragmented α-amylase@UiO-66 could be due to the tetrahedral cavities of the MOF (i.e., window pore) with 0.7 nm diameter [36] (Table 1), which is only slightly larger than the α-helix diameter (~0.6 nm). Thus, it is possible that some α-helix polypeptide chains carried by the fragmented α-amylase molecule could be infiltrated into the tetrahedral window pore due to the strong host-guest interaction and size-matching effect. Meanwhile, the window pores from MIL-101(Cr) (0.86 nm) [37], MIL-100(Al) (0.48 and 0.86 nm for pentagonal and hexagonal windows, respectively) [38,40], and MIL-53(Al) (0.85 nm) [39] are too small or too large compared to α-helix chains, suggesting an inadequate host-guest interaction between the enzyme and MOF.

In addition to the size-matching effect between the α-helix chain and the window of UiO-66, the α-helix polypeptide chains that entered the MOF micropores possibly formed hydrogen bonds with the organic linker or possess chelating interaction with zirconium metal ions which may enhance the host-guest effect, further stabilizing the retention of fragmented α-amylase on the microporous MOF.

However, the fragmented α-amylase molecules immobilized on MIL-101(Cr), MIL-100(Al), or MIL-53(Al) were only based on hydrogen bond and noncovalent interactions, such as dipole–dipole or Van der Waals forces between the peptide bonds and the organic linkers. Thus, the reusability of the fragmented α-amylase will be low and prone to leakage after several catalytic cycles leading to the decreased catalytic performances (Table 1).

To fully utilize the application of the developed bioreactors toward the hydrolysis of starch, several parameters including the enzyme concentration, immobilization time, and reaction temperature were optimized. For instance, the best catalytic efficiency was obtained when lower amounts of enzyme were immobilized onto the UiO-66 as depicted in Figure S8. Further increase in enzyme concentration led to a decrease in catalytic efficiency, which could be due to enzyme aggregation or autolysis. In a separate condition, the immobilization time may also affect the amount of enzyme loaded onto the UiO-66. Thus, the amount of time to immobilize the enzyme was evaluated from 30, 60, and 90 min (Figure S9). As shown, poor catalytic performance was obtained when the immobilization time was carried out at 90 min, suggesting the leakage of the fragmented α-amylase, while lowering to 60 min further improved its catalytic efficiency. The obtained enzyme loading capacity based on the change in fluorescence intensity of fragmented α-amylase solution at 328 nm (before and after immobilization in MOFs) was demonstrated to be 99.7 μg/mg carrier for UiO-66 (Figure S10).

**Figure 2:**
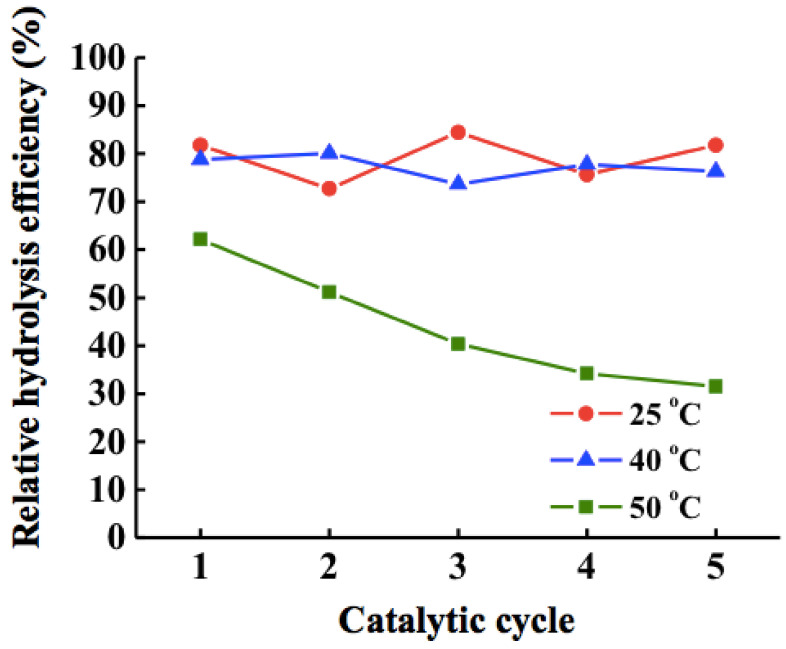
Comparison of the starch hydrolysis efficiency of fragmented α-amylase@UiO-66 at different reaction temperature.

Apart from the immobilization time and the amount of enzyme, the reaction temperature was also optimized to enhance the performance of the bioreactors. It was observed that increasing the reaction temperature from 25 °C to 50 °C significantly affects the catalytic efficiency of the fragmented α-amylase@UiO-66, which dramatically decreases from 80% to 60% hydrolysis efficiency (Figure 2). This result could be ascribed to the heat lability of amylase, leading to its deactivation as the temperature is further increased to 50 °C. Collating these results, it shows that the immobilized fragmented α-amylase@UiO-66 is economically viable and low cost, thus, could be useful as a bioreactor to be utilized for the hydrolysis of starch. Besides, batch-to-batch development of the bioreactor shows consistent results based on the obtained catalytic performances, suggesting the reliability of the proposed carrier-enzyme system (Table S2). To verify that the catalytic activity is from the fragmented α-amylase@UiO-66 and not from the carrier, blank determination for the catalytic efficiency of UiO-66 on starch was carried out, showing no catalytic activity. The reaction did not exhibit conversion of starch to maltose, as seen in the UV-VIS experiment (Figure S11).

Considering the long-term use of the bioreactor, we further explored its stability for one month. As shown, the catalytic performance of the immobilized fragmented α-amylase@UiO-66 reactor demonstrated stable hydrolysis efficiency for 15 cycles and can be useful for one month without any significant loss in the catalytic activity of the immobilized enzyme (Figure S12). Interestingly, this result is comparable or even better than the previous reported α-amylase immobilized bioreactors, which requires complex surface functionalization prior to the immobilization of enzymes (Table S3). In terms of economical and sustainability point of view, we can prepare the bioreactors in a faster time without the need for surface modification, thus, decreasing the total cost in preparing the bioreactor.

## 4. Conclusions

To summarize, this work demonstrated a simple strategy to immobilize the fragmented protein α-amylase into microporous MOFs to afford several bioreactors. Among them, the fragmented α-amylase@UiO-66 demonstrated efficient and stable catalytic performance for the hydrolysis of starch, due to proper size matching of the α-helices of the enzyme fragments with the pore size of the MOF. Furthermore, the proposed strategy of immobilizing the fragmented α-amylase showed promising potential through simple host-guest interaction and also features economically viable and eco-friendly synthesis processes due to the absence of any surface modification on the solid supports. Finally, this strategy would also be beneficial for future studies, especially in catalysis and drug delivery systems.

## Figures and Tables

**Table 1:** Window/channel size, BET surface area, and catalytic efficiency (%) of fragmented α-amylase on metal-organic frameworks (MOFs) supports.

MOFs	Window/Channel Size (nm)	BET Surface Area (m^2^ g^−1^)	Catalytic Efficiency (%) ^[a]^	
			1st Catalysis	2nd Catalysis
UiO-66	0.7 [36]	1060	91.6	82.0
MIL-101(Cr)	0.86, 1.2, 1.47, 1.6 [37]	2452	68.6	63.1
MIL-100(Al)	0.48, 0.58, 0.86 [38]	1300	76.8	52.7
MIL-53(Al)	0.85 [39]	1353	53.6	26.4

^[a]^ (Catalytic efficiency of fragmented α-amylase@MOF/catalytic efficiency of in-solution fragmented α-amylase) × 100%.

## Data Availability

Data is contained within the article and the supplementary material.

## Supplementary Materials

The following are available online at https://www.mdpi.com/1996-1944/14/4/870/s1: Part I: Synthesis and characterization of materials, which includes the experimental parameters on how characterization was carried out. Part II: Application, which includes additional experimental data as well as comparison to other α-amylase bioreactors found in literature.
