# Supplementary material for: Fragmented α-Amylase into Microporous Metal-Organic Frameworks as Bioreactors
Source: Materials (Basel). 2021 Feb 11;14(4):870. doi: 10.3390/ma14040870 (PMC7918099; doi:10.3390/ma14040870)
Supplement: Supplementary file 1 [file materials-14-00870-s001.zip › materials-1090075-supplementary.pdf]

materials

Supplementary

Fragmented α-Amylase into Microporous Metal-Organic Frameworks as Bioreactors

Li-Hao Liu [1,†], Ru-Yin Chiu [1,†], Pamela Berilyn So [1], Stephen Lirio [1], Hsi-Ya Huang [1,‡], Wan-Ling Liu [1] and Chia-Her Lin [2,*]

[1] Department of Chemistry, Chung Yuan Christian University, Taoyuan 32023, Taiwan; leedorins@gmail.com (L.-H.L.); jo6yplum@gmail.com (R.-Y.C.); pbtiuso@gmail.com (P.B.S.); stephenblirio@gmail.com (S.L.); hyhuang@cycu.edu.tw (H.-Y.H.); starsea800@hotmail.com (W.-L.L.)
[2] Department of Chemistry, National Taiwan Normal University, Taipei 11676, Taiwan
* Correspondence: chiaher@ntnu.edu.tw
† These authors contributed equally to this work.
‡ Deceased August 22, 2017.

Table of Contents

Part I. Synthesis and Characterization

1. Synthesis of UiO-66
2. Synthesis of MIL-101(Cr)
3. Synthesis of MIL-53(Al)
4. Synthesis of MIL-100(Al)
5. Experimental Parameters for the Instruments

Part II. Application

1. Figure S1
2. Figure S2
3. Figure S3
4. Figure S4
5. Figure S5
6. Figure S6
7. Figure S7
8. Figure S8
9. Figure S9
10. Figure S10
11. Figure S11
12. Figure S12
13. Table S1
14. Table S2
15. Table S3

Part I Synthesis and Characterization

1. Synthesis of UiO-66

UiO-66 was synthesized and activated according to published procedures.[1] Synthesis of UiO-66 was performed by dissolving $ZrCl_4$ (0.053 g, 0.227 mmol) and 1,4-benzenedicarboxylic acid ($C_8H_6O_4$, 1,4-BDC) (0.034 g, 0.227 mmol) in N,N′-dimethylformamide (DMF) (24.9 g, 340 mmol) at room temperature (RT). The obtained mixture was sealed and placed in a pre-heated oven at 120 °C for 24 h. After cooling in air to RT, the resulting solid was filtered, repeatedly washed with DMF, and dried at RT.

2. Synthesis of MIL-101(Cr)

$[Cr_3O(BDC)_3(F)(H_2O)_2]\cdot 25H_2O$ was synthesized and activated according to published procedures.[2] Generally, MIL-101(Cr) was hydrothermally synthesized from a mixture of chromium nitrate nonahydrate ($Cr(NO_3)_3\cdot 9\ H_2O$) (400 mg, 1.0 mmol), 1,4-BDC (166 mg, 1.0 mmol), hydrofluoric acid (HF) (0.2 mL) and H_2O (5 mL) placed in a 23 mL Teflon autoclave. The mixture was heated at 220 °C for 8 h. The resulting green powdered sample was collected by filtration, washed with purified water and ethanol (EtOH), then dried at RT. Activation was done by further heating for 1 day in DMF then stirring in EtOH overnight. The MIL-101(Cr) placed under vacuum and heated at 150 °C for 1 day before further experiments.

3. Synthesis of MIL-53(Al)

MIL-53(Al) was synthesized and activated according to published procedures.[3] The starting materials $Al(NO_3)_3\cdot 9H_2O$ (1.30 g, 1 mmol), 1,4-BDC (0.288 g, 0.5 mmol) and H_2O (80 mmol), were placed in a Teflon autoclave and heated at 220 °C for 72 h in a conventional oven (Thermo Fisher, Waltham, MA, USA). The resulting powder sample was collected by filtration and washed with deionized water (D.I. water). The solid was purified upon heating in air at 330 °C for 3 days to evacuate unreacted and the occluded 1,4-BDC molecules contained in the structure.

4. Synthesis of MIL-100(Al)

A mixture of $Al(NO_3)_3\cdot 9H_2O$ (0.6640 g, 1.8 mmol), trimethyl-1,3,5-benzenetricarboxylate (Me_3BTC, 0.3783 g, 1.5 mmol), nitric acid (HNO_3) (0.3 mL, 4 mmol), and H_2O (5 mL) were placed in a Teflon autoclave and was heated synthesized with the aid of microwave (MLS GmbH, Auenweg, Germany) at 200 °C for 30 min. The powder was filtered off and washed with DMF at 150 °C for 4 h. The powder was then soaked in H_2O and stirred at 80 °C for 12 h. Finally, the MIL-100(Al) was placed under vacuum and heated at 110 °C for 1 day before further experiments.[4]

5. Experimental Parameters for the Instruments

UV-Vis spectrometer

All UV-Vis experiments were performed using Shimadzu system (UV-2550, Shimadzu, Tokyo, Japan). The scanning wavelength was set from 450 to 650 nm and the absorption maxima were observed at 518 nm. The slit width was set at 1.0 nm.

Fluorescence spectrometer

All fluorescence experiments were performed using Hitachi system (F-7000, Hitachi, Tokyo, Japan). The fluorescence scan was performed in emission mode with the following conditions: a) scan speed of 1200 nm/min, excitation wavelength of 278 nm, excitation slit width of 5 nm and b) emission wavelength from 288 nm to 540 nm, emission slit width of 10 nm and detection wavelength of 328 nm.

Circular dichroism (CD) spectrometer

All CD experiments were performed using AVIV system (Model 410, Aviv Biomedical Inc., New Jersey, NJ, USA). The wavelength was set at 190–260 nm, the average detection time was 10 s /1.0 nm, and the number of scan was 2.

Scanning electron microscope (SEM)

SEM images were obtained from a JEOL system (JSM-7600F, JEOL, Tokyo, Japan) and the applied voltage was set at 10 kV. The samples were dried in a vacuum system for one day and then placed on the sample holder covered with carbonized tape prior to analysis.

Powder X-ray diffraction (PXRD)

The X-ray diffraction (XRD) patterns of all the materials were recorded using Bruker D8 Advance ECO diffractometer (Bruker AXS, Karlsruhe, Germany) equipped with a Cu anode and a Cu K_α radiation source with wavelength of 1.5406 Å.

Mass spectrometry (MS)

All MS experiments were performed in a amazon SL system (Bruker AXS, Karlsruhe, Germany). The following parameters are as follows: a) ionic mode: positive; b) end plate offset: −500 V; c) nebulizer pressure: 0.55 bar; d) drying gas flow: 5.0 L/min; e) drying gas temp: 220 °C; f) detection mass range: 50-1000 (m/z); g) sample inject flow rate: 220 μL/h. For sample preparation, the hydrolyzed products were diluted with ethanol (1:1000; v/v) prior to analysis.

Part II Application

1.

Circular dichroism (CD) spectroscopy in the far-UV region (190–250 nm) was used to characterize the presence of α-helix secondary structure in various α-amylase solutions. CD spectra show two characteristic shapes for α-helix conformations (two negative bands at 208 nm and 220 nm and a positive band at 190 nm), and the intensity of the band was highly relative to the amounts of α-helix chains.[5] Results indicated that α-amylase was immersed in 6 M urea solution (Tris buffer), urea molecules caused a serious interference to the observation of the band at 190 nm, but the change in the negative band intensity at 208 nm and 222 nm clearly confirmed a higher amount of α-helix chains present in urea solution.

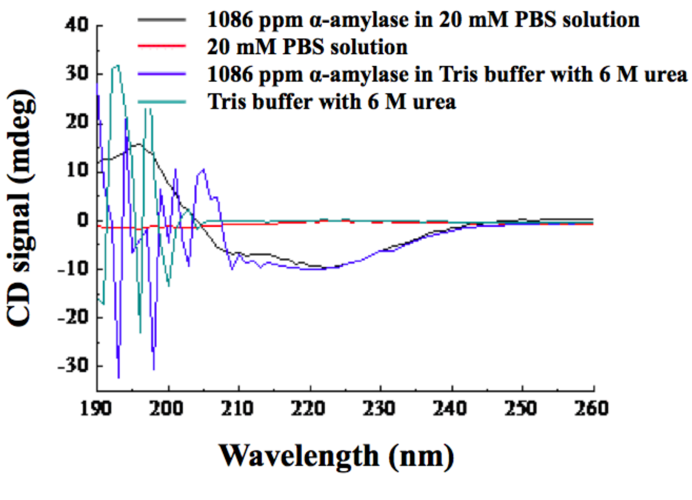

Figure S1. CD spectra of α-amylase with different preparations.

2.

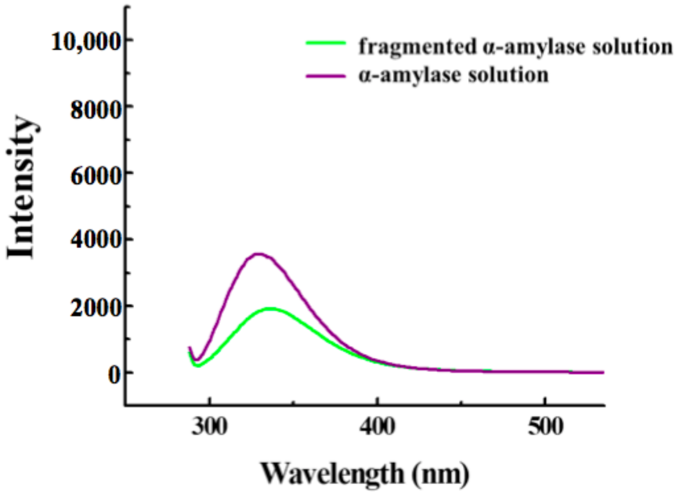

(**a**)

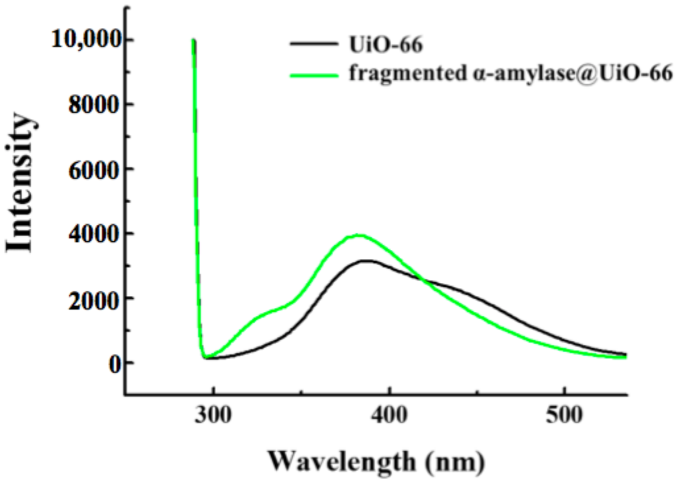

(**b**)

Figure S2. Fluorescence spectra of (**a**) native and fragmented α-amylase solution; (**b**) as synthesized UiO-66, and fragmented α-amylase@UiO-66.

3.

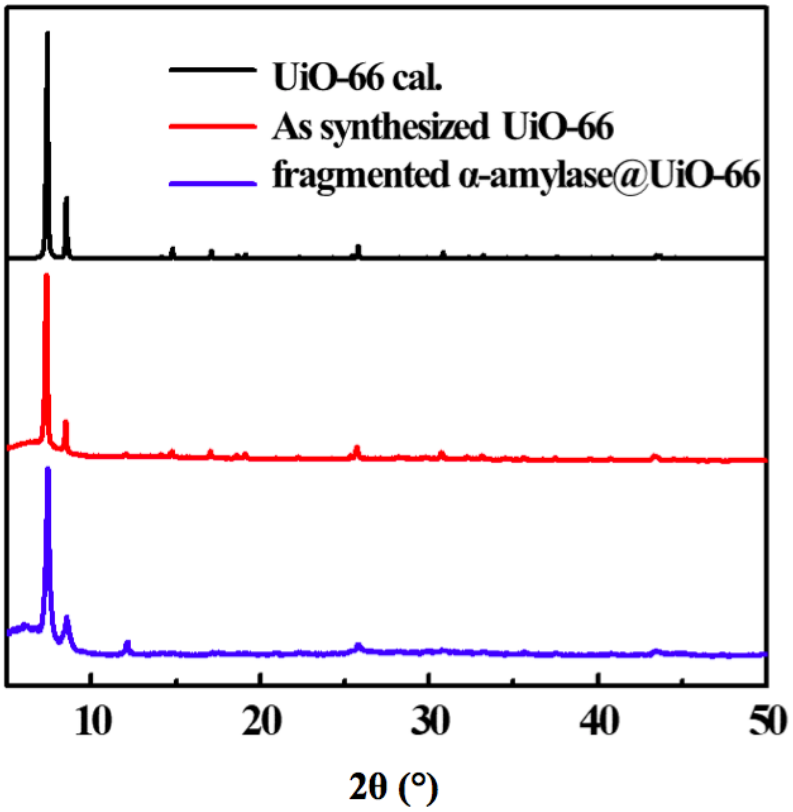

Figure S3. PXRD patterns of as synthesized UiO-66 and fragmented α-amylase@UiO-66.

4.

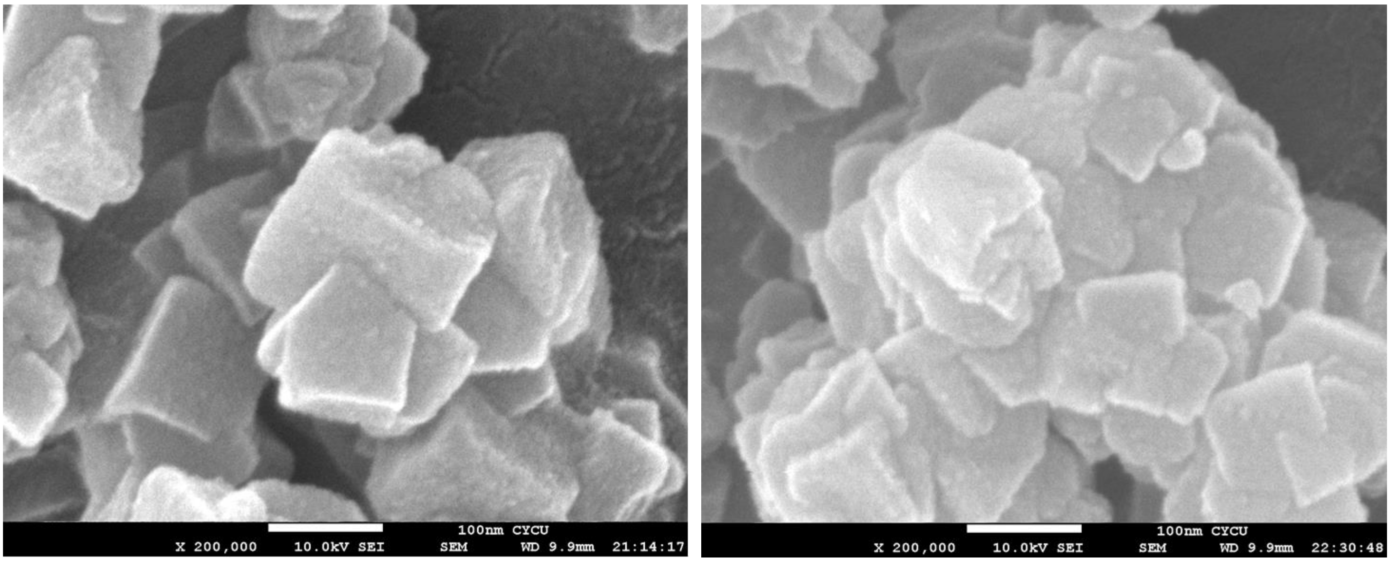

(**a**) (**b**)

Figure S4. SEM images of (**a**) as synthesized UiO-66, and (**b**) fragmented α-amylase@UiO-66.

5.

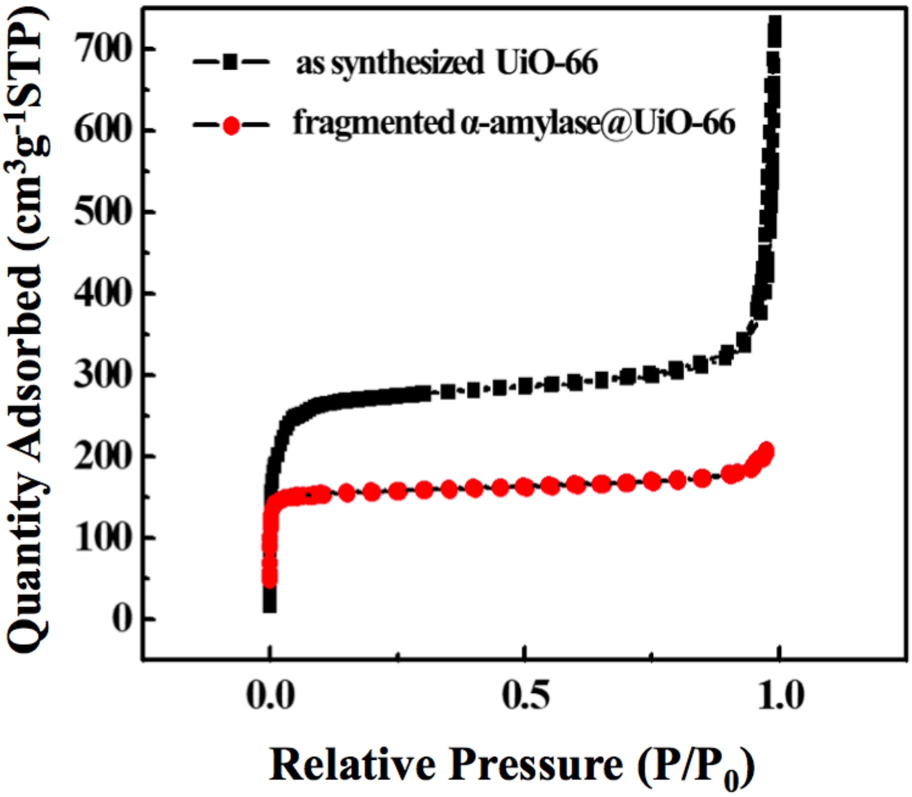

Figure S5. The nitrogen adsorption and desorption isotherms (77 K) of as-synthesized UiO-66 and fragmented α-amylase@UiO-66.

6.

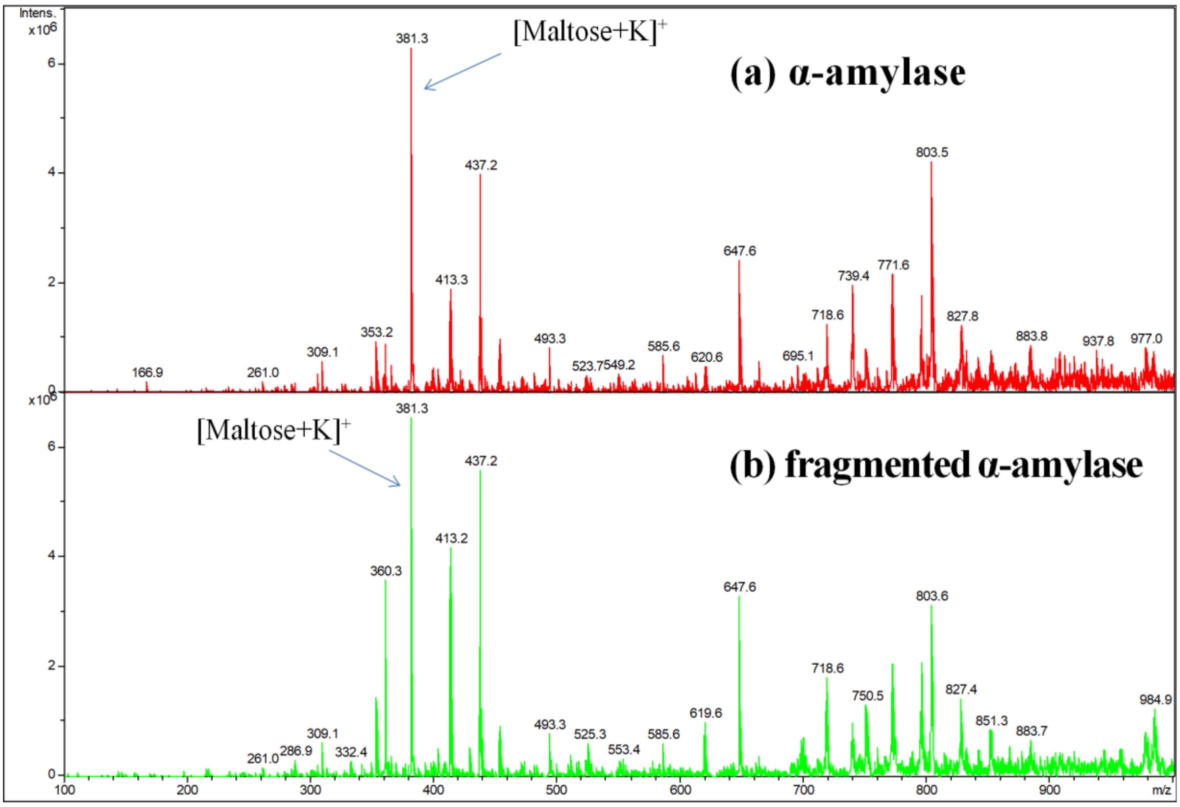

Figure S6. Mass spectrum for identifying starch hydrolysis product (positive ion mode).

7.

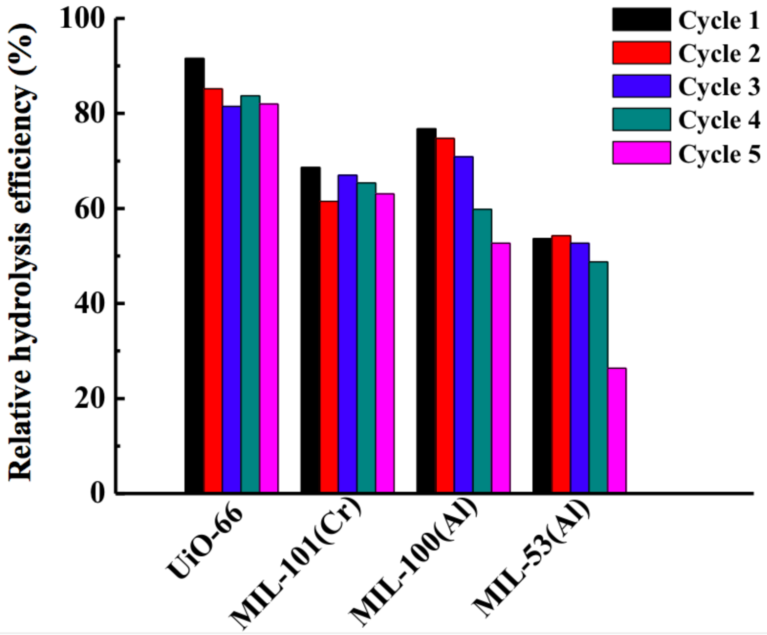

Figure S7. The hydrolytic performance of fragmented α-amylase immobilized on different MOFs.

8.

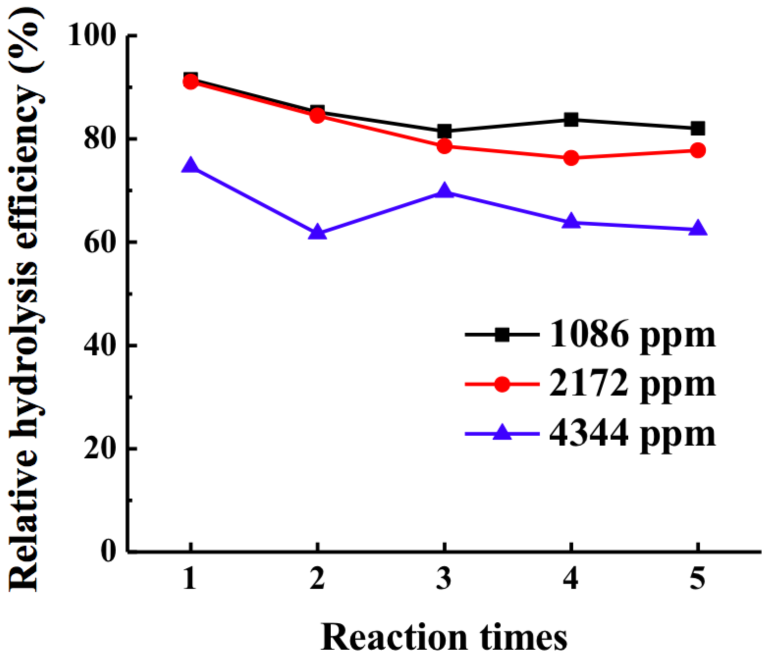

Figure S8. Comparison of the starch hydrolysis efficiency of fragmented α-amylase@UiO-66 at different enzyme concentrations.

9.

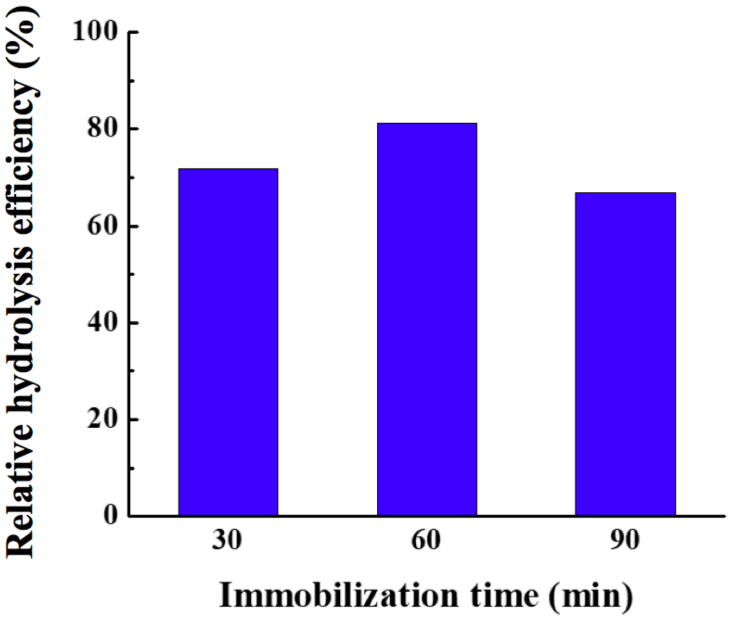

Figure S9. Comparison of the starch hydrolysis efficiency of fragmented α-amylase@UiO-66 at different immobilization times.

10.

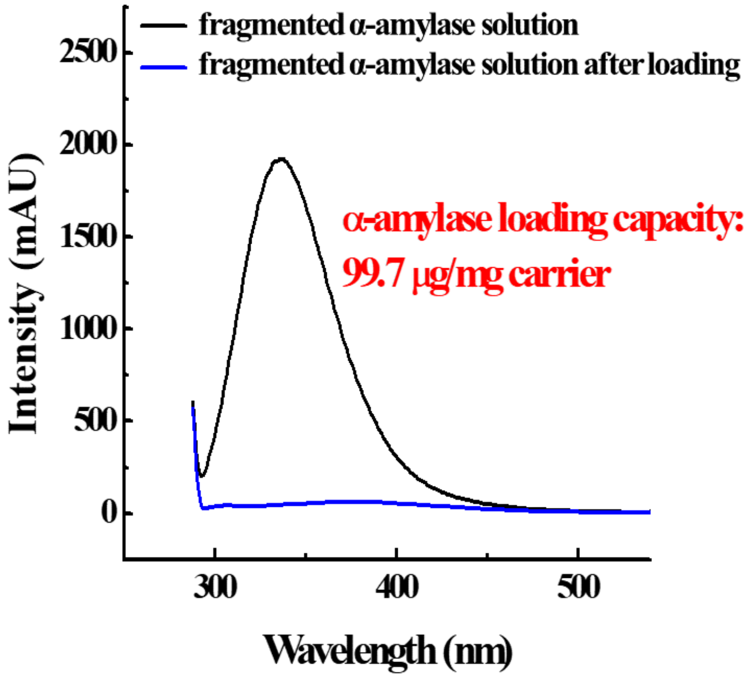

Figure S10. The loading capacity of UiO-66 for fragmented α-amylase evaluated using fluorescence emission of the fragmented α-amylase solution before and after loading.

11.

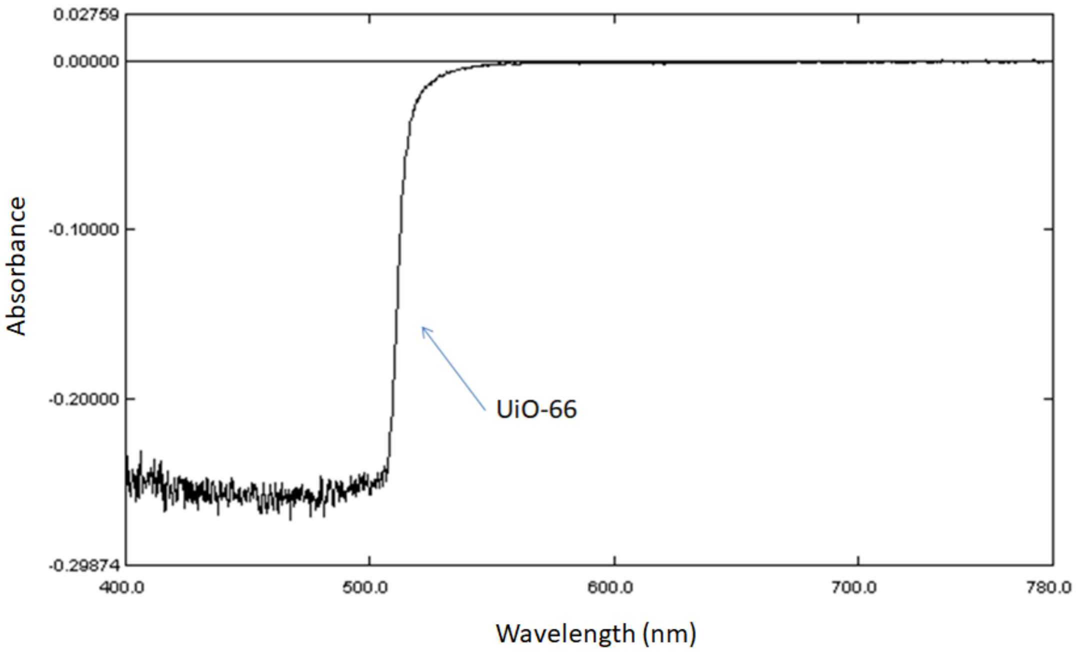

Figure S11. UV-Vis spectra of blank determination for UiO-66 catalytic activity for starch hydrolysis.

12.

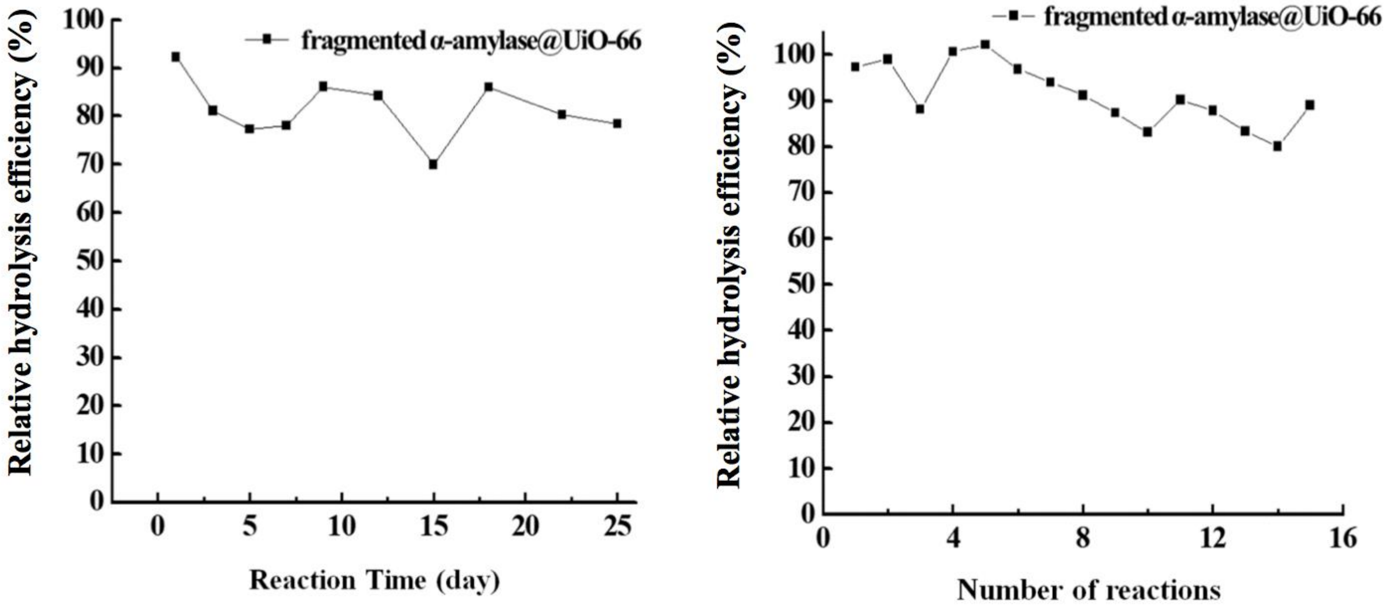

(**a**) (**b**)

Figure S12. Stability of fragmented α-amylase@UiO-66 towards the hydrolysis of starch solution (**a**) cycle-to-cycle and (**b**) day-to-day.

13.

Table S1. Surface area of as-synthesized UiO-66 and fragmented α-amylase@UiO-66.

Material	BET surface area (m^2/g)
as-synthesized UiO-66	1060
fragmented α-amylase@UiO-66	41.8

14.

Table S2. Comparison for the starch hydrolysis efficiency of batch-to-batch fragmented α-amylase@UiO-66.

Relative abs. (%)	Fragmented α-amylase@UiO-66				
	batch 1	batch 2	batch 3	avg.	RSD (%)
Cycle 1	82.00	88.77	91.56	87.44	5.62
Cycle 2	88.94	86.77	85.18	86.96	2.17
Cycle 3	89.52	82.63	81.48	84.54	5.14
Cycle 4	90.04	87.89	83.72	87.22	3.68
Cycle 5	96.19	86.84	82.01	88.35	8.16

15.

Table S3. Literatures of α-amylase bioreactors.

Reference	Substrate	Surface modification	Procedure of α-amylase immobilization	Stability and efficiency of bioreactor
[6]	magnetite nanoparticles	(1) functionalized by 3-aminopropyltriethoxy silane (APTS): 2 h<br>(2) reacted with 2,4,6-trichloro-1,3,5-triazine: 4.5 h	covalent bonding: 25 °C, 4 h	Reuse: 6 cycle (85.22%)<br>Day to day: 12 day (79.99%)
[7]	polymer	-	covalent bonding: 25 °C, 24 h	Reuse: 15 cycle (76.52%)<br>Day to day: 30 day (86.7%)
[8]	polymer nanofibers	activation: 40 °C, 24 h	covalent bonding: 25 °C, 12 h	Reuse: 15 cycle (80%)<br>Day to day: 30 day (82.9%)
[9]	polymer/Ag nanocomposites	activation: 4 °C	covalent bonding: 25 °C, 12 h	Reuse: 10 cycle (80%)
[10]	magnetic nanoparticles	APTS-functionalized: 40 °C, 2 h	physical adsorption: 45 °C, 1 h	Reuse: 4 cycle (~80%)
[11]	polymer	functionalized by glutaraldehyde and dry: 2-3 h	cross linking: 25 °C, 24 h	Reuse: 15 cycle (85%)<br>Day to day: 3 months (60%)
**This work**	**MOFs (UiO-66)**	**-**	**fragmentation: 3 h**<br>**physical adsorption: 25 °C, 1 h**	**Reuse: 15 cycle (~80%)**<br>**Day to day: 25 days (80%)**

References

1. Cavka, J.H.; Jakobsen, S.; Olsbye, U.; Guillou, N.; Lamberti, C.; Bordiga, S.; Lillerud, K.P. A New Zirconium Inorganic Building Brick Forming Metal Organic Frameworks with Exceptional Stability. *J. Am. Chem. Soc.* **2008**, *130*, 13850–13851, doi:10.1021/ja8057953.
2. Férey, G.; Mellot-Draznieks, C.; Serre, C.; Millange, F.; Dutour, J.; Surblé, S.; Margiolaki, I. A Chromium Terephthalate-Based Solid with Unusually Large Pore Volumes and Surface Area. *Science* **2005**, *309*, 2040–2042, doi:10.1126/science.1116275.
3. Loiseau, T.; Serre, C.; Huguenard, C.; Fink, G.; Taulelle, F.; Henry, M.; Bataille, T.; Férey, G. A Rationale for the Large Breathing of the Porous Aluminum Terephthalate (MIL-53) Upon Hydration. *Chem.—Eur. J.* **2004**, *10*, 1373–1382, doi:https://doi.org/10.1002/chem.200305413.
4. Volkringer, C.; Popov, D.; Loiseau, T.; Férey, G.; Burghammer, M.; Riekel, C.; Haouas, M.; Taulelle, F.; Synthesis, Single-Crystal X-ray Microdiffraction, and NMR Characterizations of the Giant Pore Metal-Organic Framework Aluminum Trimesate MIL-100. *Chem. Mater*. **2009**, *21*, 5695–5697, doi:10.1021/cm901983a.
5. Sreerama, N.; Venyaminov, S.Y.U.; Woody, R.W. Estimation of the number of α-helical and β-strand segments in proteins using circular dichroism spectroscopy. *Protein Sci.* **1999**, *8*, 370–380, doi:https://doi.org/10.1110/ps.8.2.370.
6. Sohrabi, N.; Rasouli, N.; Torkzadeh, M. Enhanced stability and catalytic activity of immobilized α-amylase on modified Fe3O4 nanoparticles. *Chem. Eng. J.* **2014**, *240*, 426–433, doi:https://doi.org/10.1016/j.cej.2013.11.059.
7. Emrah, C.; Ozkan, D.; Serap, D.; Yusuf, M. Alpha-Amylase Immobilization on Epoxy Containing Thiol-Ene Photocurable Materials. *J. Microbiol. Biotechnol.* **2013**, *23*, 205–210, doi:10.4014/jmb.1209.09017.
8. Baştürk, E.; Demir, S.; Danış, Ö.; Kahraman, M.V. Covalent immobilization of α-amylase onto thermally crosslinked electrospun PVA/PAA nanofibrous hybrid membranes. *J. Appl. Polym. Sci.* **2013**, *127*, 349–355, doi:https://doi.org/10.1002/app.37901.
9. Khan, M.J.; Husain, Q.; Ansari, S.A. Polyaniline-assisted silver nanoparticles: A novel support for the immobilization of α-amylase. *Appl. Microbiol. Biotechnol*. **2013**, *97*, 1513–1522, doi:10.1007/s00253-012-4384-6.

10. Zhang, Q.; Han, X.; Tang, B. Preparation of a magnetically recoverable biocatalyst support on monodisperse Fe3O4 nanoparticles. *RSC Advances* **2013**, *3*, 9924–9931, doi:10.1039/C3RA40192H.
11. Singh, S.; Saikia, J.P.; Buragohain, A.K. A novel reusable PAni-PVA-Amylase film: Activity and analysis. *Colloids Surf. B. Biointerfaces* **2013**, *106*, 46–50, doi:https://doi.org/10.1016/j.colsurfb.2013.01.031.
